# Identification of Chitinolytic Enzymes in *Chitinolyticbacter meiyuanensis* and Mechanism of Efficiently Hydrolyzing Chitin to *N*-Acetyl Glucosamine

**DOI:** 10.3389/fmicb.2020.572053

**Published:** 2020-10-20

**Authors:** Alei Zhang, Xiaofang Mo, Ning Zhou, Yingying Wang, Guoguang Wei, Zhikui Hao, Kequan Chen

**Affiliations:** ^1^State Key Laboratory of Materials-Oriented Chemical Engineering, College of Biotechnology and Pharmaceutical Engineering, Nanjing Tech University, Nanjing, China; ^2^Taizhou Vocational and Technical College, Taizhou, China

**Keywords:** *Chitinolyticbacter meiyuanensis* SYBC-H1, complete genome sequencing, chitinolytic enzymes, chitin, *N*-acetyl glucosamine

## Abstract

*Chitinolyticbacter meiyuanensis* SYBC-H1, a bacterium capable of hydrolyzing chitin and shrimp shell to *N*-acetyl glucosamine (GlcNAc) as the only product, was isolated previously. Here, the hydrolysis mechanism of this novel strain toward chitin was investigated. Sequencing and analysis of the complete genome of SYBC-H1 showed that it encodes 32 putatively chitinolytic enzymes including 30 chitinases affiliated with the glycoside hydrolase (GH) families 18 (26) and 19 (4), one GH family 20 β-*N*-acetylglucosaminidase (NAGase), and one Auxiliary Activities (AA) family 10 lytic polysaccharide monooxygenase (LPMO). However, only eight GH18 chitinases, one AA10 LPMO, and one GH20 NAGase were detected in the culture broth of the strain, according to peptide mass fingerprinting (PMF). Of these, genes encoding chitinolytic enzymes including five GH18 chitinases (*Cm*711, *Cm*3636, *Cm*3638, *Cm*3639, and *Cm*3769) and one GH20 NAGase (*Cm*3245) were successfully expressed in active form in *Escherichia coli*. The hydrolysis of chitinous substrates showed that *Cm*711, *Cm*3636, *Cm*3638, and *Cm*3769 were endo-chitinases and *Cm*3639 was exo-chitinase. Moreover, *Cm*3639 and *Cm*3769 can convert the GlcNAc dimer and colloidal chitin (CC) into GlcNAc, which showed that they also possess NAGase activity. In addition, NAGase *Cm*3245 possesses a very high exo-acting activity of hydrolyzing GlcNAc dimer. These results suggest that chitinases and NAGase from SYBC-H1 both play important roles in conversion of *N*-acetyl chitooligosaccharides to GlcNAc, resulting in the accumulation of the final product GlcNAc. To our knowledge, this is the first report of the complete genome sequence and chitinolytic enzyme genes discovery of this strain.

## Introduction

Chitin, a β-1,4-linked *N*-acetyl glucosamine (GlcNAc) polymer, is the second most abundant polysaccharide in nature with production of 100 billon tons per year (Kaur and Dhillon, 2015). GlcNAc prepared from chitin exhibits specific bioactivities that have been used widely in various fields such as dietary supplements, cosmetic ingredients, and osteoarthritis therapeutics (Chen et al., 2010; Liu et al., 2013). Thus, the conversion of abundant chitin biomass to valuable GlcNAc is both economically and ecologically significant. At present, GlcNAc production from chitin on an industrial scale relies mainly on the hydrolysis of strong acids, which lead to low yield and acidic wastes during the synthesis procedure (Zhang et al., 2016b). Thus, enzymatic production of GlcNAc from chitin using chitinolytic enzymes is a better approach because it is generally environmentally friendly, produces high yields, and generates GlcNAc with high bioactivities (Zhang et al., 2018a).

The degradation of chitin to GlcNAc requires cooperation of chitinolytic enzymes such as chitinase [mainly belonging to glycoside hydrolase (GH) families 18 and 19; hydrolyzes chitin to *N*-acetyl chitooligosaccharides (*N*-acetyl COSs)]; β-N-acetylglucosaminidase (GH family 20; hydrolyzes *N*-acetyl COSs to GlcNAc); and lytic polysaccharide monooxygenase (LPMO) [Auxiliary Activity (AA) families 10 and 11; cleavage of chitin chains with oxidation to enhance the hydrolysis of chitin] (Zhang et al., 2018b). Many microbes have been identified as inexpensive sources of chitinolytic enzymes. However, most reported crude chitinolytic enzymes always produce a mixture of GlcNAc and *N*-acetyl COSs during enzymatic hydrolysis of chitin. For example, the crude enzymes derived from *Trichoderma* sp. and *Bacillus* sp. produce GlcNAc of 36 mg and (GlcNAc)_2_ of 28 mg from 200 mg of chitin (Kudan et al., 2010). The crude enzymes from *Bacillus licheniformis* SK-1 chitinase completely hydrolyzed chitin within 6 days, resulting in 75% GlcNAc and 20% (GlcNAc)_2_ (Pichyangkura et al., 2002). To date, there are few reports of crude chitinolytic enzymes capable of hydrolyzing chitin to produce GlcNAc as the only product (Sashiwa et al., 2002).

A Gram-negative bacterium *Chitinolyticbacter meiyuanensis* SYBC-H1 belonging to the family *Neisseriaceae* was isolated previously (Hao et al., 2011). Crude chitinolytic enzymes secreted by SYBC-H1 efficient degrade chitin and shrimp shells to GlcNAc without *N*-acetyl COSs (Zhang et al., 2016b; Wei et al., 2017). However, the specific mechanism is still unknown. In this study, the complete genome of SYBC-H1 was sequenced and genes encoding potential chitinolytic enzymes were analyzed. Furthermore, the key chitinolytic enzymes in culture broth of SYBC-H1 were determined based on the results of peptide mass fingerprinting (PMF). The genes encoding key chitinolytic enzymes were heterologous expressed in *Escherichia coli*. In addition, the activity and hydrolysis pattern of these recombinant chitinolytic enzymes were also investigated. This study will help us understand the mechanism by which chitinolytic enzymes from SYBC-H1 convert chitin to GlcNAc as the sole product and advance potential biotechnological applications of SYBC-H1.

## Materials and Methods

### Strains, Plasmids, and Chemicals

*Chitinolyticbacter meiyuanensis* SYBC-H1 [CGMCC 3438 T, ATCC BAA-2140(T)] was previously isolated from a soil sample at Meiyuan Park (33°56′N, 120°18′E), Wuxi city, China (Hao et al., 2011). *E. coli* DH5α and *E. coli* BL21(DE3) (Novagen Co., Shanghai, China) were, respectively, used in sub-cloning and expression of chitinolytic enzyme genes from *C. meiyuanensis* SYBC-H1. *E. coli* strains were cultivated in Luria-Bertani (LB) broth or on agar plates containing 50 μg/mL kanamycin. The pMD19-T and pET-28a(+) plasmids were, respectively, used as the subclone and expression vector of the chitinolytic enzyme genes.

Chitin, GlcNAc, *p*-nitrophenyl *N*-acetyl glucosaminide (*p*NP-GlcNAc), *p*-nitrophenyl *N,N*′-diacetyl chitobiose [*p*NP-(GlcNAc)_2_], and *p*-nitrophenyl *N*,*N*′,*N*″-triacetyl chitotriose [*p*NP-(GlcNAc)_3_] were purchased from Sigma–Aldrich Co. Ltd. (Shanghai, China). The *N*-acetyl chitooligosaccharides [(GlcNAc)_2_-(GlcNAc)_6_] were acquired from Qingdao BZ Biotechnology Co., Ltd. (Qingdao, China). Peptone and yeast extract were purchased from the Oxoid Co., Ltd. (Beijing, China). The molecular reagents were purchased from Takara Bio Inc. (Dalian, China). Colloidal chitin (CC) was prepared as described in a previous study (Gao et al., 2015). Other chemicals used in this study were purchased from local suppliers at analytical grade or higher purity.

### Complete Genome Sequencing and Assembly

SYBC-H1 was cultured according to our previous studies (Zhang et al., 2016a, 2018b). The genomic DNA was extracted using the TIANamp Bacteria DNA Kit (Tiangen Biotech Co., Ltd., Beijing, China). The full genome was sequenced by Personal Biotechnology Co., Ltd. (Shanghai, China). A whole genome shotgun strategy was used in this study. The libraries of different genome inserts (0.2-67 kb) were constructed and sequenced using the third generation single-molecule sequencing technology on the PacBio sequencer. The data obtained by PacBio were assembled using HGAP4 (Chin et al., 2016) and CANU (V1.6) (Koren et al., 2017) software to obtain scaffold sequences. Contigs obtained by splicing the second-generation and third-generation sequencing data were collinearly analyzed using MUMmer (v3) (Delcher et al., 1999) software, the assembly results were confirmed again and the contigs were determined. The positional relationship between the contigs was filled with gaps. Finally, the results were corrected using pilon (version 1.22) (Walker et al., 2014) and then spliced to obtain the complete sequence.

### Genome Annotation and Analysis

Sequence alignment of protein-coding genes was performed using DIAMOND (v0.9.10.111) software (Buchfink et al., 2015) based on the National Center for Biotechnology Information (NCBI^1^) nr database. Swiss-Prot^2^, Kyoto Encyclopedia of Genes and Genomes (KEGG^3^), and Clusters of Orthologous Groups (COG^4^) databases were used for functional annotation. Pairwise BLAST-based Average Nucleotide Identity (ANI) were obtained using JSpecies^5^ and digital DNA–DNA Hybridization (dDDH) values were calculated with the Genome-to-Genome Distance Calculator 99 (GGDC 2.1), using formula 2^6^. The KO and Pathway annotations of the protein-coding genes were mainly performed using KEGG’s automated annotation system (KAAS Ver. 2.1) (Moriya et al., 2007). The promoter was analyzed using Promoter 2.0 Prediction Server^7^. Nucleotide and amino acid sequences were analyzed using Snap Gene^TM^ 1.1.3 software^8^ and the ExPASy ProtParam tool^9^. The Carbohydrate-Active enZyme (CAZy) enzyme genes present in the genomic sequence were predicted using hmmscan (3.1b2, February 2015) (Yoon, 2009) software. Enzyme locations were analyzed using the SignalP 5.0 server^10^ and Gneg-mPLoc server v.2.0^11^. The conserved domains and classifications were identified using SMART database^12^. The DNA and protein sequence alignments were performed via the NCBI server with the programs BLASTN and BLASTP^13^, respectively.

### Identification of Chitinolytic Enzymes in Fermentation Broth of *C. meiyuanensis* SYBC-H1

The production of chitinolytic enzymes of the strain was conducted according to our previous study (Zhang et al., 2018b). The supernatant from the culture was used for PMF analysis with an electrospray ionization quadrupole time-of-flight mass spectrometer (ESI-Q-TOF MS/MS) technique (PROTTECH, Inc., Suzhou, China). The detected peptide masses were then compared to theoretical mass values in the Mascot website databases^14^ to reveal the amino acid sequences of the peptide fragments. The peptide sequence was then aligned with the genome of *C. meiyuanensis* SYBC-H1 (GenBank accession number, CP041335) to find the corresponding proteins and their coding genes.

### Cloning and Expression of Genes Encoding Chitinolytic Enzymes in Culture Broth

The genomic DNA of SYBC-H1 was used as the template for PCR amplification. Primers with suitable restriction sites designed to amplify the genes are listed in Table 1. The primers were used to amplify DNA fragments corresponding to the whole ORF without any putative signal peptide. The amplified PCR products were purified by gel electrophoresis, digested with restriction enzymes, and then ligated into pMD19-T vector and sequenced by Invitrogen Corporation (Shanghai, China). The positive recombinant plasmids were digested with corresponding restriction enzymes, and these genes were inserted into the pET-28a(+) vector expression plasmid with a C-terminal His6 tag. The recombinant plasmids were introduced into *E. coli* BL21 (DE3) and then cultured in LB medium (containing 50 μg/mL kanamycin) at 37°C in a shaker with a rotation speed of 200 r/min. When the cell density (OD_600_) was 0.6, isopropyl β-D-thiogalactoside was added at a final concentration of 1 mM for protein induction, and was further grown at 25°C for 12 h.

**TABLE 1:** Oligonucleotide primers used for PCR in this study.

**Primers**	**Oligo-nucleotides (5′-3′)**	**Restriction sites**
*ORF711-F*	GAATTCCATATGCGTTCCCG GCCTGGCAG	*Nde*I
*ORF711-R*	TCCGCTCGAGCTGCAGGGTA GCGGCCG	*Xho*I
*ORF3245-F*	GAATTCCATATGATGAGCCG TCCCGCCGGATC	*Nde*I
*ORF3245-R*	TCCGCTCGAGGGCGCCCACC TGCACCG-3′	*Xho*I
*ORF3636-F*	GAATTCCATATGGCCCATGC CGCACCGGCATG	*Nde*I
*ORF3636-R*	CCCAAGCTTGTACTTCAACG CGCTGCAG	*Hin*dIII
*ORF3638-F*	GAATTCCATATGGCTGAATG GGCGGAAGGCAAC	*Nde*I
*ORF3638-R*	TCCGCTCGAGGTGTGATGTC CGGCGTACGC	*Xho*I
*ORF3639-F*	GAATTCCATATGCGTACCCG GTATGGCAGG	*Nde*I
*ORF3639-R*	TCCGCTCGAGCATGGGCAAC ATGAACAAG	*Xho*I
*ORF3769-F*	GAATTCCATATACCGAGATC GCCCCGTACTTC	*Nde*I
*ORF3769-R*	TCCGCTCGAGGGTGCTGGGC AACGGCCGC	*Xho*I

*The underlined sequences are restriction enzyme cutting sites.*

The cells were harvested by centrifugation at 6000 *g* and 4°C for 5 min, after which the cell precipitation was re-suspended with His-tag binding buffer (20 mM Tris-HCl, 500 mM NaCl, 50 mM imidazole [pH 7.0]) and lyzed by JY92-IIN ultrasonication (Ningbo Xinzhi biotechnology, Ltd., Ningbo, China). Cell debris was removed by centrifugation at 8000 *g* for 5 min and the supernatant was purified using a fast protein liquid chromatography (FPLC) system (GE AKTA Pure 150; General Electric Co., Boston, MA, United States) with a Ni-nitrilotriacetic acid affinity chromatography column (His Trap FF 5 mL). The target protein was eluted with elution buffer (20 mM Tris-HCl, 500 mM NaCl, 250 mM imidazole [pH 7.0]). The eluted fractions were passed through 10 kDa ultrafiltration tubes (Millipore, United States) to remove the imidazole with sodium phosphate (pH 7.0) and concentrate the recombinant enzymes.

The purified enzymes were analyzed by sodium dodecyl sulfate-polyacrylamide gel electrophoresis (SDS-PAGE) with a 3% stacking gel and a 10% separating gel, according to the method described by Laemmli (1970). The protein marker (Takara Biotechnology Co., Ltd., Nanjing, China) containing 180-, 140-, 100-, 75-, 60-, 45-, 30-, and 25-kDa proteins was used as the molecular mass standard. Protein concentrations were determined using the Bradford method with bovine serum albumin as the standard (Bradford, 1976).

### Determination of Enzyme Activities

The assay of enzyme activities used CC and chromogenic substrates. For CC, a mixture containing 0.5 mL CC of 1% (w/v), 1.4 mL 50 mM sodium phosphate buffer (pH 7.0), and 100 μg enzyme, was incubated for 30 min at 37°C. After incubating at 37°C for 30 min, the reaction was terminated by boiling for 5 min. Reducing sugars released during the reaction were quantified using GlcNAc standards at concentrations ranging from 0.25–1.25 μmol/mL, according to the 3,5-dinitrosalicylic acid method. For (GlcNAc)_2_, the standard reaction mixture (1 mL) contained 50 mM sodium phosphate buffer (pH 7.0), 10 g/L (GlcNAc)_2_, and 5 μg enzyme. After incubation at 37°C for 30 min, the reaction was terminated by boiling for 5 min. The GlcNAc released was measured by liquid chromatography (LC). One unit (1 U) of chitinase activity was defined as the amount of enzyme required to produce 1 μmol GlcNAc per minute at 37°C.

For chromogenic substrates, *p*NP-GlcNAc, *p*NP-(GlcNAc)_2_, and *p*NP-(GlcNAc)_3_ were used as substrates, the standard reaction mixture (1 mL) contained 50 mM sodium phosphate buffer (pH 7.0), 0.5 mM chromogenic substrate, and 5 μg enzyme. The mixture was incubated at 37°C for 30 min, and then the reaction was terminated by adding 1 mL of 0.2 M NaOH. The amount of *p*NP released during the reaction was measured by reading the optical density at 405 nm. One unit of chitinase activity was defined as the amount of enzyme required to release 1 μmol *p*NP from the substrate per minute at 37°C.

### The Hydrolysis Pattern of Chitinolytic Enzymes Toward CC

Reaction mixtures (1 mL) containing purified chitinolytic enzymes (50 μg) and 0.5 mL CC at a final concentration of 10 g/L were incubated in 0.4 mL of 50 mM sodium phosphate buffer (pH 7.0) at 37°C for 12 h. In each case, the supernatant after hydrolysis was centrifuged at 12,000 *g* for 5 min. Reaction products were analyzed using an Agilent 1260 series LC system (Agilent Technologies, Santa Clara, CA, United States) according to our previous study (Zhang et al., 2018b).

### Nucleotide Sequence Accession Number

The complete genome sequence of *C. meiyuanensis* SYBC-H1 was deposited in GenBank under the accession number CP041335.

## Results and Discussion

### General Features and Gene Annotation of the Complete Genome of *C. meiyuanensis* SYBC-H1

To explore the genetic basis for the ability of chitin degradation, the complete genome of strain *C. meiyuanensis* SYBC-H1 was sequenced. The genome shows the highest similarity to the reported genome of *Chitiniphilus shinanonensis* strain SAY3 (95.85%) (Sato et al., 2009), followed by *Chitinibacter tainanensis* DSM15459 (94.58%) (Chern et al., 2004). ANI and dDDH values were used to accurately measure nucleotide-level genomic similarity between SYBC-H1 and the two strains. The results showed that the ANI and dDDH values between SYBC-H1 and SAY3 genomes were 78.73 and 22.30%, respectively. The ANI and dDDH values between the SYBC-H1 and DSM15459 genomes were 71.09 and 19.20%, respectively. These ANI and dDHH results are far lower than the species cut-off thresholds of 96 and 70% for ANI and dDDH, respectively, suggesting that SYBC-H1 is not the same species as SAY3 or DSM15459.

**FIGURE 1:**
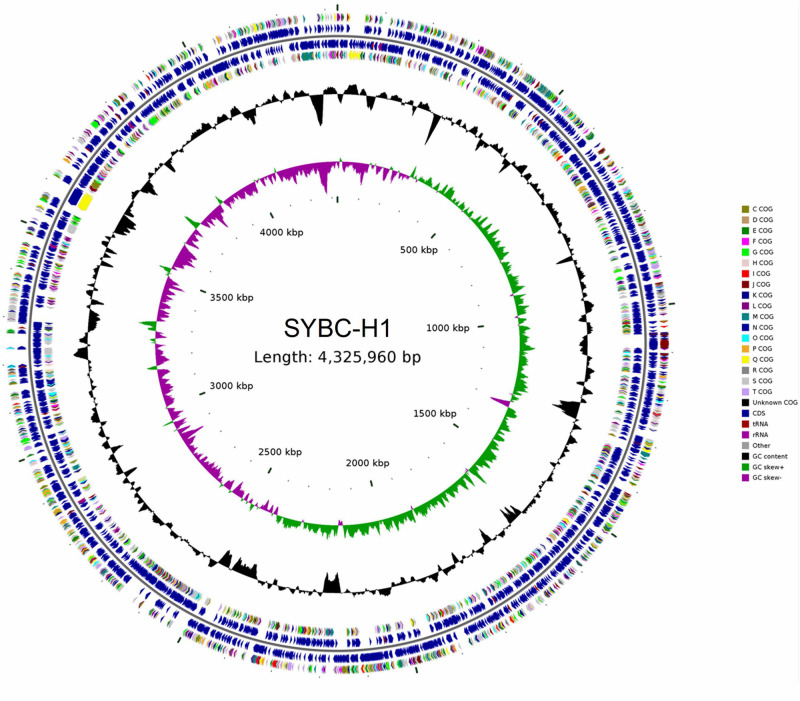
Classical circular genome maps of the *C. meiyuanensis* SYBC-H1. From the inner circle to the outermost, each circle represents (1) genome size (0.5 Mb/scale), (2) GC skew, (3) GC content reverse CDS, (4) COG groups of each CDS, (5) CDS, (6) rRNA/tRNA.

The characteristics of the complete genome of SYBC-H1 are shown in Table 2. The final assembly of the SYBC-H1 genome was 4.3 Mb in size with a high GC content of 63.52%, 57 tRNA genes, 12 rRNA genes, and three other ncRNA genes (Figure 1 and Table 2). These data show some differences from SAY3 and DSM15459 genomes.

**TABLE 2:** Genome features of *C. meiyuanensis* SYBC-H1.

**Features**	**SYBC-H1**	**SAY3**	**DSM15459**
Genome size (Mb)	4.33	4.15	3.42
GC content (%)	63.5	66.4	56.1
proteins	4,018	3,658	3,141
ORF total length (bp)	3,815,517	–	–
ORF average length (bp)	928.8	–	–
Longest ORF length (bp)	37,698	–	–
ORF density	0.949 genes per kb	–	–
tRNAs	56	54	54
rRNAs (5S, 16S, 23S)	12	6	13
ncRNAs	3	4	4
GenBank accession number	CP041335	ARGN00000000	AUCN00000000

A total of 4108 ORFs, which account for 88.20% of the total nucleotides, were obtained in the SYBC-H1 genome. Among these genes, 3734 (90.9%), 3436 (83.64%), 1932 (47.03%), 2691 (65.51%), and 2838 (69.08%) coding genes were functionally annotated using NR (NCBI), COG, KEGG, Swiss-Prot, and Gene Ontology (GO) databases, respectively (Table 3).

**TABLE 3:** The functional annotation of protein coding gene.

**Annotation in database**	**No. of genes**	**Proportion of annotation (%)**
NR	3,734	90.90
COG	3,436	83.64
KEGG	1,932	47.03
Swiss-Prot	2,691	65.51
GO	2,838	69.08

### Prediction and Analysis of Genes Encoding Chitinolytic Enzymes in the SYBC-H1 Genome

**FIGURE 2:**
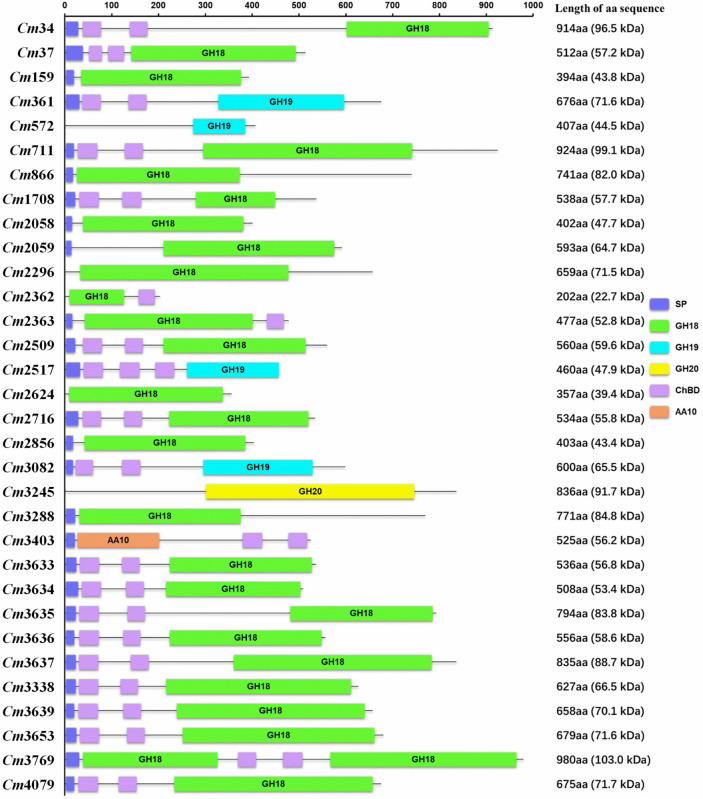
Domain organization of chitinolytic enzymes. Protein domains were identified using SMART databases. aa, amino acid; SP, signal peptide; GH18, Glycoside Hydrolase family 18; GH19, Glycoside Hydrolase family 19; GH20, Glycoside Hydrolase family 20; ChBD, chitin-binding domain; AA10, Auxiliary Activity family 10; Black line, no function area.

Bacteria utilize chitin as a carbon source by degrading it into GlcNAc, a process that requires synergy of several chitinolytic enzymes such as chitinase and NAGase (Bhattacharya et al., 2007). Analysis of the SYBC-H1 genome showed that it encodes 32 enzymes that could potentially be involved in chitin utilization, including 26 GH18 chitinases, four GH19 chitinases, one GH20 β-*N*-acetyl glucosaminidase (NAGase), and one AA10 LPMO (Figure 2), which is more than most bacteria such as SAY3 (12 GH18 chitinases, one GH19 chitinase, and one GH20 NAGase) (Huang et al., 2012a), *Streptomyces coelicolor* A3 (11 GH18 chitinases, two GH19 chitinases, and five exo-NAGases) (Bentley et al., 2002), *Paenibacillus xylanilyticus* W4 (six GH18 chitinases) (Liao et al., 2019). The 32 chitinolytic enzymes, except for *Cm*3769 with two GH18 catalytic domains, have only one catalytic domain. Chitin-binding domain (ChBD) can efficiently anchor the domains on the surface of chitin and help catalytic domains to digest insoluble chitin efficiently (Boraston et al., 2004). Except for GH 18 chitinases (*Cm*2058, *Cm*2059, and *Cm*2362) and GH20 NAGase (*Cm*3245), most of them contain one or two ChBDs, which is similar to most reported chitinolytic enzymes (Huang et al., 2012a; Yang et al., 2016; Lee et al., 2018; Tanaka et al., 2018).

SAY3, the most similar bacterium to SYBC-H1, encodes 15 chitinolytic enzymes (ChiA–O), consisting of 12 GH18 chitinases, one GH19 chitinase, one GH20 family NAGase, and a protein with only two ChBDs and no catalytic domains (ChiF). Seven genes encoding GH18 ChiB–H form a cluster in the SAY3 genome. Of these, five genes encoding GH18 ChiC–G are likely to constitute an operon, as well as the genes encoding GH18 ChiL and ChiM (Huang et al., 2012a,b). A similar result was found in the SYBC-H1 genome, with seven genes encoding GH18 chitinases *Cm*3633-3639 likely to form a cluster, as well as genes encoding GH18 chitinases *Cm*2058-2059 and genes encoding chitinases GH18 *Cm*2362-2363. Analysis of the operon identified short distances in ORF3635-3638 (74–177 bp) and ORF2058-2059 (174 bp). Moreover, the promoter-like sequence was also identified upstream of the ORF3638 and ORF2058. The results showed that ORF3635-3638 likely form an operon as well as ORF2058-2059.

### Identification of Chitinolytic Enzymes in Culture Broth of *C. meiyuanensis* SYBC-H1

Although we identified 32 genes encoding chitinolytic enzymes in the SYBC-H1 genome, the expression patterns of these genes in medium containing chitin is not known. Thus, the chitinolytic enzymes in culture broth of SYBC-H1 were identified by combining PMF and genomic data. As shown in Table 4, 10 chitinolytic enzymes containing eight GH18 chitinases (*Cm*711, *Cm*3635, *Cm*3636, *Cm*3637, *Cm*3638, *Cm*3639, *Cm*3653, and *Cm*3769), one AA10 LPMO (*Cm*3403), and one GH20 NAGase (*Cm*3245) were determined in culture broth. Among them, the relative contents of *Cm*711 (10.2%), *Cm*3245 (5.2%), *Cm*3636 (12.4%), *Cm*3638 (8.9%), *Cm*3639 (19.3%), and *Cm*3769 (11.3%) in broth were high, which showed that they play a key role in degrading chitin to GlcNAc. BLAST analyses revealed that these 10 chitinolytic enzymes showed significant similarity (60.54–99.70%) to various bacterial chitinolytic enzymes. However, their enzymatic characteristics have not been clarified.

**TABLE 4:** Chitinolytic enzymes in culture broth of *C. meiyuanensis* SYBC-H1.

**Chitinolytic enzymes**	**Relative content**	**Probability**	**Family**	**^a^Molecular Weight (kDa)**	**Signal peptide**	**The closest relative sequence (similarity)**	**Accession number**
*Cm*711	10.2%	99.0%	GH18	96.8	Yes	*Chitinilyticum litopenaei* chitinase (81.06%)	WP_028454508
*Cm*3245	5.2%	99.0%	GH20	91.7	No	*Chitiniphilus shinanonensis* NAGase (81.68%)	WP_018749679
*Cm*3403	3.6%	99.0%	AA10	53.7	Yes	*Iodobacter* sp. BJB302 chitinase (60.54%)	WP_099398589
*Cm*3635	1.3%	99.0%	GH18	81.1	Yes	*Chitinibacter* sp. ZOR0017 chitinase (74.24%)	WP_047395740
*Cm*3636	12.4%	99.0%	GH18	55.9	Yes	*Andreprevotia lacus* DSM23236 chitinase (62.95%)	SMC28638
*Cm*3637	3.9%	99.0%	GH18	84.9	Yes	*Jeongeupia* sp. USM3 chitinase (77.83%)	WP_070528194
*Cm*3638	8.9%	99.4%	GH18	63.7	Yes	*Chitiniphilus eburneus* chitinase (78.41%)	WP_136773598
*Cm*3639	19.3%	99.0%	GH18	67.7	Yes	*Staphylococcus* sp. J2 chitinase (99.70%)	AGC59908
*Cm*3653	0.2%	88.3%	GH18	68.8	Yes	*Chitiniphilus eburneus* chitinase (78.62%)	WP_136774653
*Cm*3769	11.3%	99.0%	GH18	100.7	Yes	*Chitiniphilus eburneus* chitinase (74.52%)	WP_136773598

*^*a*^Molecular weight without signal peptide.*

Bacteria need to degrade chitin to GlcNAc before they can use it. However, the substrate chitin has a large molecular weight and cannot enter cells. Thus, bacterial chitinolytic enzymes must be secreted extracellularly to utilize chitin. Prediction of signal peptides showed that eight GH18 chitinases and one AA10 LPMO possess a putative N-terminal signal peptide, which showed that the locations of these enzymes were extracellular. To date, most reported NAGases (hydrolyze the *N*-acetyl COSs to GlcNAc) are secretory proteins (Konno et al., 2012; Zhou et al., 2017; Visnapuu et al., 2020). However, there was no putative signal peptide in the sequence of GH20 NAGase *Cm*3245. On the contrary, the prediction using Gneg-mPLoc 2.0 showed that the location of *Cm*3245 was in the periplasm. In addition, *Cm*3245 was detected in the fermentation broth of SYBC-H1. This phenomenon suggests that *Cm*3245 is a secretory protein.

Our previous study showed that chitinolytic enzymes purified from the culture broth of SYBC-H1 based on the affinity adsorption between ChBD and substrate chitin can also hydrolyze chitin to GlcNAc as the sole product (Zhang et al., 2016b). However, NAGase *Cm*3245 cannot be purified in this process, because it does not have ChBD. Therefore, we infer that other detected chitinolytic enzymes (besides *Cm*3245) may also possess NAGase activity.

### Cloning and Expression of Genes Encoding Key Chitinolytic Enzymes

**FIGURE 3:**
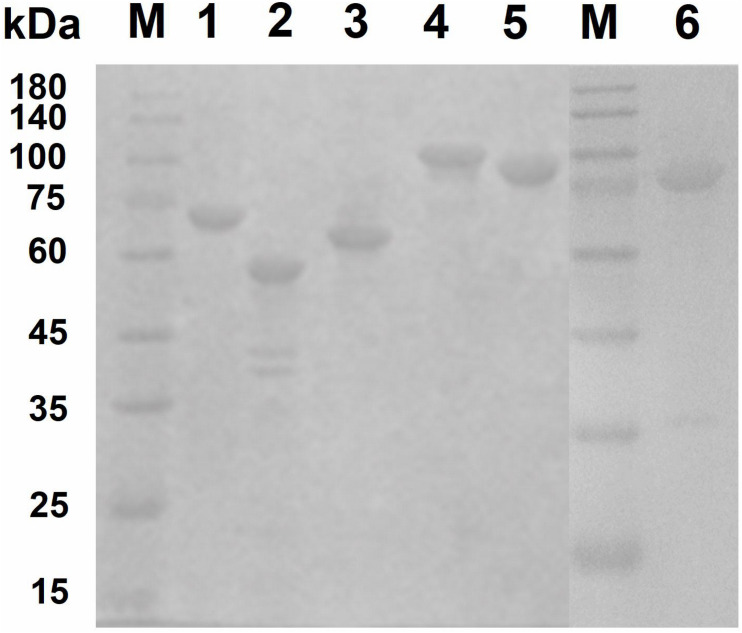
SDS–PAGE analysis of purified recombinant proteins. The purified recombinant proteins (20 μg) were analyzed by SDS-PAGE using a 3% stacking gel and a 10% separating gel with addition of SDS. Lane M, molecular weight markers; lane 1, *Cm*3639; lane 2, *Cm*3636; lane 3, *Cm*3638; lane 4, *Cm*3769; lane 5, *Cm*711; lane 6, *Cm*3245.

To investigate the key enzymes (*Cm*711, *Cm*3245, *Cm*3636, *Cm*3638, *Cm*3639, and *Cm*3769) noted above, the genes encoding these enzymes were cloned without signal peptide and expressed in *E. coli* BL21(DE3). The recombinant proteins with a C-terminal His6 tag were successfully expressed in a soluble form and were purified by Ni-NTA affinity chromatography (Figure 3). The molecular weights of recombinant *Cm*711 (96.2 kDa), *Cm*3245 (92.1 kDa), *Cm*3636 (56.3 kDa), *Cm*3638 (63.4 kDa), *Cm*3639 (67.3 kDa), and *Cm*3769 (100.1 kDa) were consistent with those calculated from the amino acid sequence. The purified recombinant proteins were used in the characterization of chitin degrading activity.

### Properties of Key Recombinant Chitinolytic Enzymes

The activities of six purified recombinant proteins were examined using CC and chromogenic substrates, *p*NP-(GlcNAc)_1–3_. As shown in Table 5, the recombinant GH18 chitinases (*Cm*711, *Cm*3636, *Cm*3638, and *Cm*3769) exhibited hydrolysis activity toward CC, as well as *p*NP-(GlcNAc)_2_ and *p*NP-(GlcNAc)_3_, which showed that they were endo-chitinases. Interestingly, *Cm*3639 and *Cm*3769 showed some hydrolysis activity toward *p*NP-GlcNAc and (GlcNAc)_2_, which revealed that they possess NAGase activity, which is similar to chitinase PbChi74 from *Paenibacillus barengoltzii* (Fu et al., 2014). In our previous study, we confirmed that *Cm*3639 (*Cm*Chi1) was a multifunctional chitinase with mainly exo-acting activity and trace endo-acting and NAGase activities, which can hydrolyze CC to only GlcNAc over a long reaction time (Zhang et al., 2018b). However, the hydrolysis products of other enzymes toward CC were unknown. Thus, the hydrolysis pattern toward CC was investigated in this study.

**TABLE 5:** Enzyme activities of recombinant key chitinolytic enzymes toward chitinous substrates.

**Enzymes**	**Enzyme activity (U/mg of protein)**				
	**CC**	**(GlcNAc)_2_**	***p*NP-GlcNAc**	***p*NP-(GlcNAc)_2_**	***p*NP-(GlcNAc)_3_**
*Cm*711	3.6 ± 0.2	nd^a^	nd	2.3 ± 0.1	1.7 ± 0.1
*Cm*3245	0.02 ± 0.001	4,233 ± 210	3,902.9 ± 130	2,140 ± 160	1,082 ± 62
*Cm*3636	2.1 ± 0.1	nd	nd	6.2 ± 0.3	0.7 ± 0.02
*Cm*3638	4.6 ± 0.2	nd	nd	8.5 ± 0.7	1.6 ± 0.1
*Cm*3639	15.3 ± 0.3	0.5 ± 0.02	0.2 ± 0.01	27.3 ± 0.6	nd
*Cm*3769	4.1 ± 0.4	13.5 ± 0.9	10.3 ± 0.6	13.3 ± 1.2	3.2 ± 0.2

*^*a*^Activity was not detected. Reaction mixture (1 mL) containing enzyme and 10 g/L CC, (GlcNAc)_2_, or 0.5 mM *p*NP-(GlcNAc)_1–3_ were incubated at 37°C and pH 7.0.*

**FIGURE 4:**
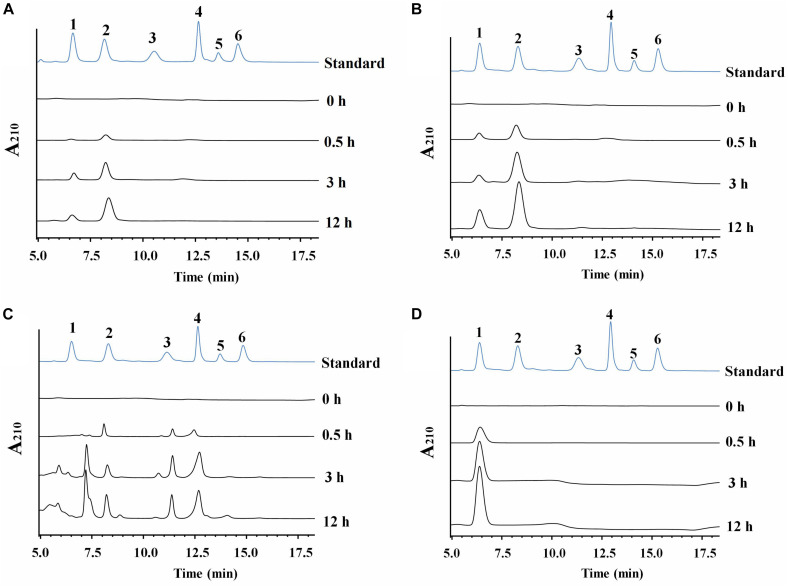
HPLC analysis of the hydrolysate of CC generated by purified recombinant chitinolytic enzymes **(A)** *Cm*711, **(B)** *Cm*3636, **(C)** *Cm*3638, and **(D)** *Cm*3769. A total of 10 g/L CC was incubated with enzymes (5 μg) in a 2 mL volume at pH 7 and 37°C for different time intervals. 1-6: Standards of GlcNAc, (GlcNAc)2, (GlcNAc)3, (GlcNAc)4, (GlcNAc)5, and (GlcNAc)6.

*Cm*711 and *Cm*3636 digested the CC into a mixture of GlcNAc and (GlcNAc)_2_ (Figure 4). *Cm*3638 mainly produces (GlcNAc)_2–4_ from CC. These results showed that *Cm*711, *Cm*3636, and *Cm*3638 possess typical characteristics of endo-type chitinases, consistent with results above. Interestingly, *Cm*3769 completely converted CC into GlcNAc as the only product from beginning to end. The result is different from *Cm*Chi1 and PbChi74, which yield mainly (GlcNAc)_2_ at the initial hydrolysis stage and (GlcNAc)_2_ needed a longer reaction time to achieve full conversion into GlcNAc. The phenomena can be explained that NAGase activity (13.5 U/mg) of *Cm*3769 is higher than its endo activity (5.1 U/mg), causing the produced *N*-acetyl COSs to be immediately degraded to GlcNAc. The result accounts for our previous study, which showed that chitinases from SYBC-H1 without NAGase degraded chitin to GlcNAc as the sole product (4). In addition, the NAGase *Cm*3245 possesses very high activity (1082-4233 U/mg) for (GlcNAc)_2_ and *p*NP-(GlcNAc)_1–3_, which showed that it represents the typical exo NAGase activity. Moreover, *Cm*3245 showed some activity (0.02 U/mg) toward CC, making it unique among NAGases. Characterization of this enzyme is ongoing.

In conclusion, NAGase activity of chitinolytic enzymes from SYBC-H1 is much higher than its chitinase activity, which leads to the accumulation of GlcNAc.

### Analysis of Genes Encoding GlcNAc Metabolic Pathway in the SYBC-H1 Genome

**FIGURE 5:**
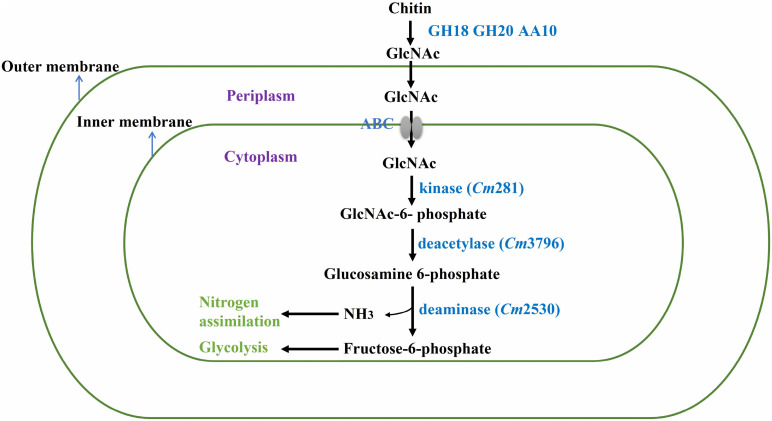
Proposed pathway of chitin degradation in *C. meiyuanensis* SYBC-H1. GH18, Glycoside Hydrolase family 18 chitinase; GH20, Glycoside Hydrolase family 20 β-*N*-acetylglucosaminidase; AA10, Auxiliary Activity family 10 lytic polysaccharide monooxygenase; ABC, ABC-sugar transporter; kinase, *N*-acetylglucosamine kinase; deacetylase, *N*-acetylglucosamine-6-phosphate deacetylase; deaminase, glucosamine-6-phosphate deaminase.

In nature, bacteria use chitin by degrading insoluble chitin into GlcNAc for their own growth (Meibom et al., 2004). Thus, the genes involved in GlcNAc metabolism in the SYBC-H1 genome were also analyzed. As shown in Figure 5, GlcNAc metabolic essential genes encoding *N*-acetylglucosamine kinase (*Cm*281, catalyzes GlcNAc to GlcNAc-6-phosphate), *N*-acetylglucosamine-6-phosphate deacetylase (*Cm*3796, catalyzes GlcNAc-6-phosphate to glucosamine 6-phosphate), and glucosamine-6-phosphate deaminase (*Cm*2530, catalyzes glucosamine 6-phosphate to fructose-6-phosphate, that enters central glycolytic pathway for cell growth) were both found (Beier and Bertilsson, 2013).

## Data Availability Statement

The datasets generated for this study can be found in the NCBI database, under BioProject PRJNA576511 and genome assemblies GCA_011149675.1, GCA_009184605.1, GCA_009183735.1, GCA_009183705.1 and GCA_009183685.1.

## Author Contributions

AZ and ZH conceived and designed the research. AZ, XM, YW, and NZ performed the experiments. AZ, XM, and GW analyzed the data. AZ and KC wrote the manuscript. All authors commented on the manuscript and approved the content.

## Conflict of Interest

The authors declare that the research was conducted in the absence of any commercial or financial relationships that could be construed as a potential conflict of interest.

## Footnotes

1 https://www.ncbi.nlm.nih.gov/genome/annotation_prok/

2 http://uniprot.org

3 http://www.genome.jp/kegg/

4 http://www.ncbi.nlm.nih.gov/COG

5 https://www.ezbiocloud.net/tools/ani

6 http://ggdc.dsmz.de/ggdc

7 http://www.cbs.dtu.dk/services/Promoter/

8 http://www.snapgene.com/

9 http://web.expasy.org/protparam/

10 http://www.cbs.dtu.dk/services/SignalP/

11 http://www.csbio.sjtu.edu.cn/bioinf/Gneg-multi/

12 http://smart.embl-heidelberg.de/

13 http://blast.ncbi.nlm.nih.gov/Blast.cgi

14 http://www.matrixscience.com
