# Supplementary figures and images for: Identification of Chitinolytic Enzymes in Chitinolyticbacter meiyuanensis and Mechanism of Efficiently Hydrolyzing Chitin to N-Acetyl Glucosamine
Source: Front Microbiol. 2020 Oct 20;11:572053. doi: 10.3389/fmicb.2020.572053 (PMC7641034; doi:10.3389/fmicb.2020.572053)

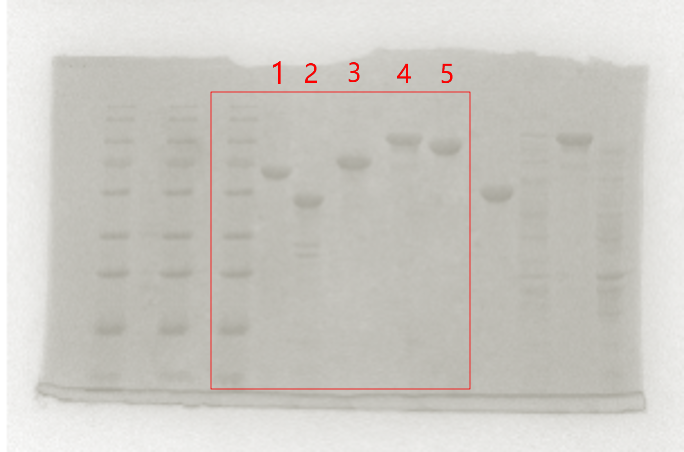

Supplement: Supplementary file 1 [file Data_Sheet_1.ZIP › Original photo1 - taged.tif]

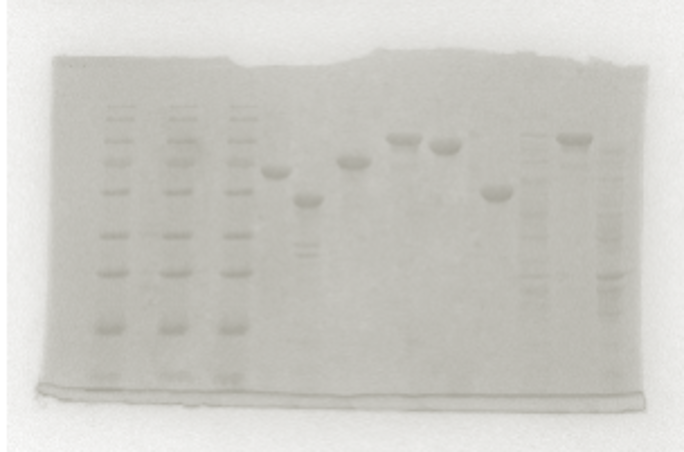

Supplement: Supplementary file 1 [file Data_Sheet_1.ZIP › Original photo1.tif]

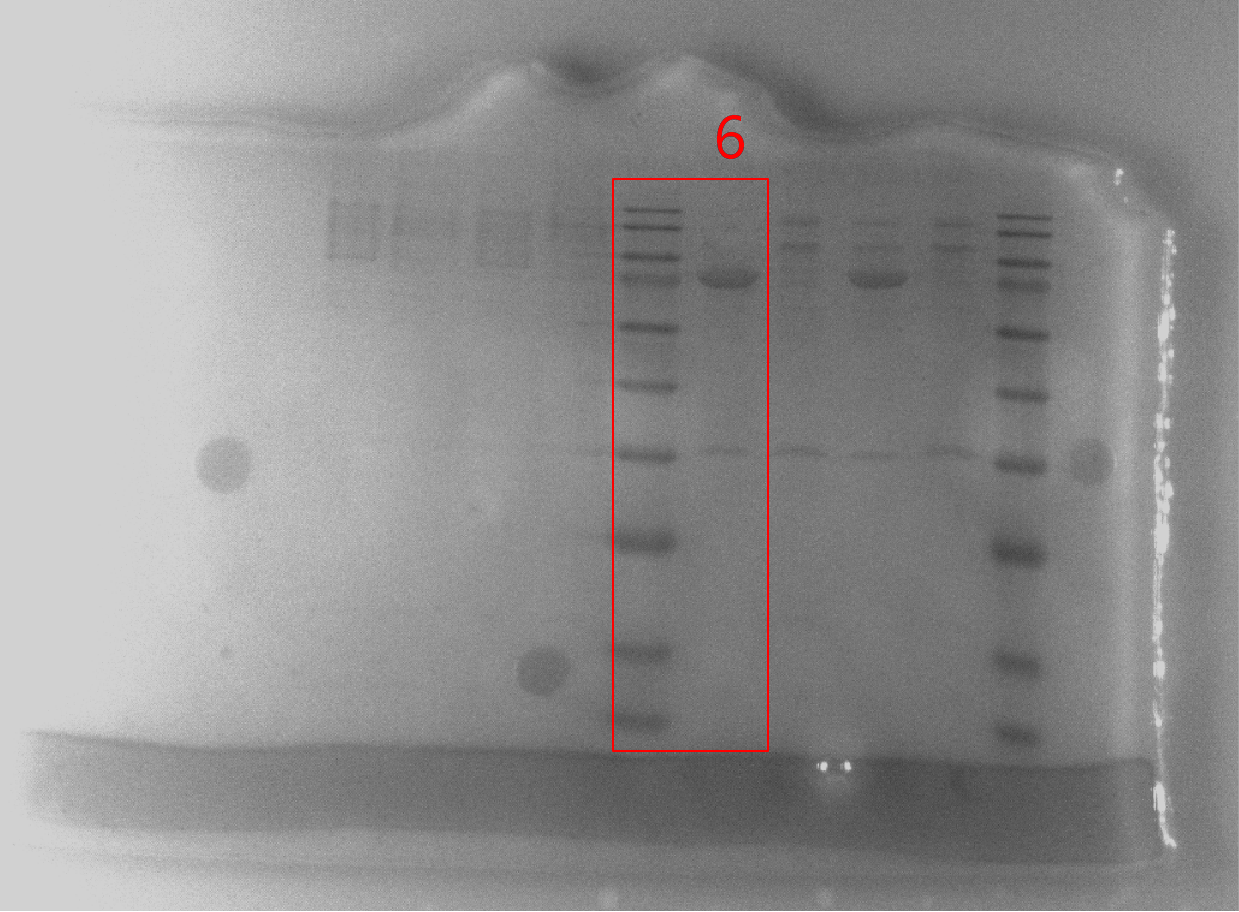

Supplement: Supplementary file 1 [file Data_Sheet_1.ZIP › Original photo2 - taged.tif]

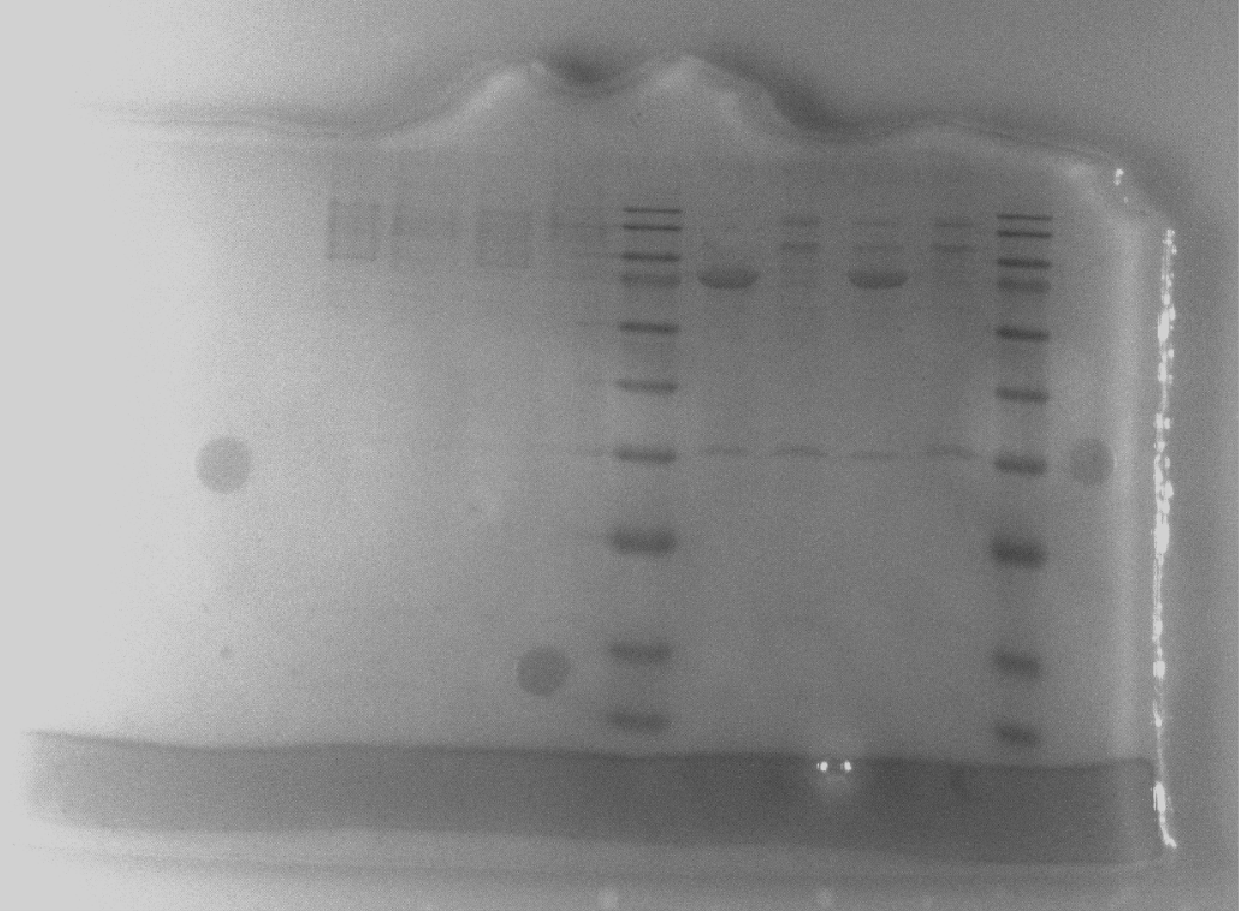

Supplement: Supplementary file 1 [file Data_Sheet_1.ZIP › Original photo2.tif]
